# Genomes of three bacteriophages from the deep subsurface aquifer

**DOI:** 10.1016/j.dib.2018.12.045

**Published:** 2018-12-18

**Authors:** Vitaly V. Kadnikov, Andrey V. Mardanov, Yulia A. Frank, Alexey V. Beletsky, Olga V. Karnachuk, Nikolai V. Ravin

**Affiliations:** a Institute of Bioengineering, Research Center of Biotechnology of the Russian Academy of Sciences, Moscow, Russia; b Faculty of Biology, Lomonosov Moscow State University, Moscow, Russia; c Laboratory of Biochemistry and Molecular Biology, Tomsk State University, Tomsk, Russia

## Abstract

Viral particles have been detected in the underground biosphere where they could be one of the main factors impacting microbial diversity, biogeochemistry and evolution. To characterize the viral component in the deep subsurface biosphere, we sequenced the metagenome of subsurface aquifer located in the Tomsk region of Russia, sampled via 2.8-km-deep borehole 5P. The *de novo* assembly of metagenomics sequences yielded three circular genomes assigned to bacteriophages of the order *Caudovirales*. The annotated genome sequences of these bacteriophages have been deposited in the GenBank database under the accession numbers MK113949, MK113950 and MK113951.

## Specifications table

**Table:** 

Subject area	Biology
More specific subject area	Metagenomics
Type of data	Genome sequences of viruses
How data was acquired	Shotgun DNA sequencing using Illumina HiSeq 2500 and MinION (Oxford Nanopore)
Data format	Analyzed complete genome sequences
Experimental factors	Complete genome sequences of three viruses were assembled from metagenome of groundwater
Experimental features	The water sample from borehole 5P, drilled to a depth of 2.8 km, was collected and then the total DNA was extracted for metagenome sequencing.
Data source location	Chazhemto village in the Tomsk region of Russia (58.0758N; 82.8374 E)
Data accessibility	Data is submitted to NCBI GenBank and it is in the public repository. The direct URL to data is https://www.ncbi.nlm.nih.gov/nuccore/MK113949;
	https://www.ncbi.nlm.nih.gov/nuccore/MK113950;
	https://www.ncbi.nlm.nih.gov/nuccore/MK113951
Related research article	None

## Value of the data

• This data provides information about genetic potential of three viruses from the deep subsurface aquifer.
• Data is applicable for comparative genomic studies of viruses of prokaryotes.
• Data will help to explore the diversity and ecological role of viruses in the deep subsurface ecosystems.

## 1 Data

Viral particles have been increasingly detected in extreme habitats including the underground biosphere. In such habitats, viruses are one of the main factors of microbial diversity, biogeochemistry and evolution [1], [2]. To determine the viral component in the underground biosphere of Western Siberia, we sequenced the metagenome of a deep subsurface aquifer located in the Tomsk region of Russia, sampled via an oil exploration borehole 5P, drilled to a depth of 2.8 km [3]. The aquifer presumably was formed in the sedimentary rocks of the Mesozoic Era. The *de novo* assembly of metagenomics sequences yielded three circular-mapping genomes assigned to the tailed bacteriophages of the order *Caudovirales*. The data in Table 1 represents genome annotation summary, including genome size, G+C content and the number of predicted genes of each bacteriophage genome.

**Table 1:** General characteristics of genome sequences of viruses.

Parameter	Phage 5P_1	Phage 5P_2	Phage 5P_3
Genome size (bp)	41,683	74,215	39,501
G + C content (%)	45.1	53.4	61.6
Predicted genes	56	105	57
Genes with assigned functions	8	21	16
GenBank accession number	MK113949	MK113950	MK113951

## 2 Experimental design, materials, and methods

### 2.1 Sample collection and preparation

Water samples were taken from a sampling line at the borehole 5P in April, 2016 [4]. Cells from 20 L of borehole water were collected on 0.22 μm cellulose nitrate membranes (Sartorius, Germany) using a Sartorius filtration unit.

### 2.2 DNA extraction

The filters were frozen in liquid nitrogen and then ground and melted with TE buffer in a water bath at 37 °C. The total DNA was extracted using Power Soil DNA Isolation Kit (MO BIO Laboratories Inc, Carlsbad, USA). About 1 μg of total DNA was isolated.

### 2.3 Sequencing and assembly

Metagenomic DNA was sequenced using the Illumina HiSeq2500 platform according to the manufacturer’s instructions (Illumina Inc.,USA). The sequencing of a paired-end (2 × 250 bp) TruSeq DNA library generated 57,579,354 read pairs. Primer and quality trimming were performed with Cutadapt v. 1.17 [5] and Sickle v. 1.33 (https://github.com/najoshi/sickle), respectively. Cutadapt was used with default settings, and Q33 score was used for Sickle. Trimmed reads were merged with FLASH v1.2.11 [6]. The same metagenomics DNA was sequenced on MinION (Oxford Nanopore), using 1D Genomic DNA by ligation protocol. 1,418,419 raw MinION reads (about 1.5 Gb in total) were *de novo* assembled into contigs using Miniasm v0.3 [7], and the assembly was polished using Racon 1.3.1 [8]. Illumina reads were mapped back to the assembled sequence using Bowtie 2 [9] and the mapping was used to obtain improved consensus sequence by Pilon 1.22 software [10].

### 2.4 Identification and annotation of viral genomes

For each of the circular contigs reported by Miniasm gene search and annotation were performed using the RAST server 2.0 [11], followed by manual correction by searching the National Center for Biotechnology Information (NCBI) databases. Circular contigs containing genes encoding phage capsid proteins were assigned to bacteriophages. All three obtained bacteriophages were classified as members of the order *Caudovirales* on the basis of sequence similarity with known phage genomes.
